# Role of Pharmacists in Hormonal Contraceptive Access: A Survey of North Carolina Pharmacists

**DOI:** 10.3390/pharmacy8040191

**Published:** 2020-10-16

**Authors:** Gwen J Seamon, Allison Burke, Casey R Tak, Amy Lenell, Macary Weck Marciniak, Mollie Ashe Scott

**Affiliations:** 1 Mountain Area Health Education Center, Asheville, NC 28803, USA; gjseamon@gmail.com; 2 Walgreens, Asheville, NC 28801, USA; allison.burke@ucdenver.edu (A.B.); amy.lenell@walgreens.com (A.L.); 3 Division of Pharmaceutical Policy and Outcomes, Eshelman School of Pharmacy, University of North Carolina at Chapel Hill, Chapel Hill, NC 27599, USA; caseytak@email.unc.edu; 4 Division of Practice Advancement and Clinical Education, Eshelman School of Pharmacy, University of North Carolina at Chapel Hill, Chapel Hill, NC 27599, USA; macary_marciniak@unc.edu

**Keywords:** contraception, women’s health, pharmacist, community pharmacy

## Abstract

The role of pharmacy in healthcare continues to evolve as pharmacists gain increased clinical responsibilities in the United States, such as the opportunity to prescribe hormonal contraception. Currently, North Carolina (NC) pharmacists do not have this ability. While previous research focused on the perceptions of community pharmacists surrounding this practice, no previous research surveyed all pharmacists in a state. This cross-sectional, web-based survey was distributed to all actively licensed pharmacists residing in the state of NC in November 2018. The primary objective was to determine the likelihood of NC community pharmacists to prescribe hormonal contraception. Secondary outcomes included: evaluation of all respondent support and perceptions of this practice as advocacy occurs on the state organization level and unified support is critical; opinions regarding over-the-counter (OTC) status of contraception; and potential barriers to prescribing. Overall, 83% of community pharmacists were likely to prescribe hormonal contraception. No differences in likelihood to prescribe were detected between geographic settings. Community pharmacists reported that the most common barriers to impact prescribing were added responsibility and liability (69.8%) and time constraints (67.2%). Fewer than 10% of respondents felt that hormonal contraception should be classified as OTC (7.9%). Noncommunity pharmacists were significantly more likely to agree that prescribing hormonal contraception allows pharmacists to practice at a higher level, that increased access to hormonal contraception is an important public health issue, and that rural areas would benefit from pharmacist-prescribed hormonal contraception. Overall, this study found a willingness to prescribe and support from the majority of both community and noncommunity pharmacists. Limitations of the study included a low response rate and potential nonresponse bias. Future research is needed to address solutions to potential barriers and uptake of this practice, if implemented.

## 1. Introduction

Approximately 54% of pregnancies in North Carolina were unintended in 2010 [1]. This has increased from 49% in 2002 and is higher than the 2011 nationwide rate of 45% [1,2]. Both the United States Department of Health and Human Services’ Healthy People 2020 and the 6/18 Initiative from the Centers for Disease Control and Prevention (CDC) include the goal of decreasing the rate of unintended pregnancies [3,4]. In accordance, North Carolina has been seeking strategies to reduce the rate of unintended pregnancies to 31.2% by 2020 through the Healthy North Carolina 2020 initiative [5]. The American College of Obstetricians and Gynecologists (ACOG) Committee on Healthcare for Underserved Women has recommended “the right of women to receive prescribed contraceptives or an immediate informed referral from all pharmacies” as one strategy to overcome these barriers [6].

Pharmacists are well-positioned to improve access to contraception. Pharmacists have the authority to prescribe hormonal contraception in a growing number of states, among which include Oregon, California, Colorado, Maryland, the District of Columbia, Hawaii, Utah, and New Mexico, and others [7,8]. Currently, pharmacists in North Carolina may prescribe medications through collaborative practice authority but are not able to prescribe or supply hormonal contraceptives without prescriptive authority [9]. Widening this scope to include pharmacist prescribing of hormonal contraception may improve women’s access to contraception and thereby reduce unintended pregnancies. Previous research exists regarding community pharmacist interest in prescribing hormonal contraception in certain states, but the support for both implementation of this service in North Carolina, and the support of all pharmacists, is currently unknown. Furthermore, no previous literature has addressed potential differences in the perceptions and support of hormonal contraceptive prescribing between community and noncommunity pharmacists. North Carolina is a largely rural state where 90 of the 100 counties were designated as Primary Care-Health Professional Shortage Areas (HPSA) in January 2020, thus the need for increased access to medications and healthcare exists [10]. Additionally, pharmacy practices and cultures vary in the United States, especially amongst geographic regions. No previous studies on the interest in pharmacist-prescribed hormonal contraception have been completed in the Southeastern region in the United States prior to this survey.

The primary objective of this study was to determine the likelihood of North Carolina community pharmacists to prescribe hormonal contraception. The secondary objectives of this study were to expand survey distribution beyond community-based pharmacists to evaluate the differences between community-based pharmacists and noncommunity pharmacists regarding perception of the potential benefits of pharmacist-prescribed hormonal contraception; assess potential barriers to prescribing reported by community-based pharmacists; stratify readiness to prescribe by geographic location; and assess pharmacists’ opinions regarding appropriate Food and Drug Administration (FDA) classification of hormonal contraception.

## 2. Materials and Methods

This cross-sectional study was conducted online via Qualtrics^®^ (Qualtrics, Provo, UT, USA), a web-based survey application, using a 26-item questionnaire. Using a database provided by the North Carolina Board of Pharmacy, a survey link was emailed to all actively licensed pharmacists residing in North Carolina. Completion of the survey was voluntary and data were deidentified. Informed consent was obtained prior to commencing the survey. Demographic information was collected for all respondents, including age, years in practice, previous pharmacy education, training and/or credentials, state of pharmacy school graduation, geographic location of primary practice site, previous education and/or training on prescribing hormonal contraceptives, and primary pharmacy practice site. Primary pharmacy practice sites were organized into two categories, those that were community-based and those that were not. Community pharmacists included those who worked in chain or retail pharmacies, community pharmacy managers, and community pharmacy owners, all others were categorized as noncommunity pharmacists. Community pharmacists were directed to additional practice site-related questions such as benefits and barriers of implementation, general attitudes toward pharmacist-prescribed hormonal contraception, personal views on prescribing hormonal contraception, and contraceptive knowledge-based questions. All other pharmacy practice sites (i.e., noncommunity pharmacy) were directed to a separate question bank regarding benefits of implementation of pharmacist-prescribed hormonal contraception, general attitudes toward pharmacist-prescribed hormonal contraception, and contraceptive knowledge-based questions.

Likert-scale questions were used to assess pharmacist opinions regarding perceived benefits and barriers to implementation of pharmacist-prescribed hormonal contraception. Survey questions were adapted from Vu et al. [11]. In order to evaluate willingness, pharmacists were surveyed to determine their readiness to prescribe and perceptions regarding prescribing hormonal contraception. Agreement to each statement was assessed with the following scale: strongly disagree, disagree, undecided, agree, or strongly agree. Comfort-related statements were assessed using the following scale: very uncomfortable, somewhat uncomfortable, neither comfortable nor uncomfortable, somewhat comfortable, and very comfortable. The survey instrument is available in Supplementary Material section.

The questionnaire was pilot-tested among a convenience sample of pharmacists for feedback on validity and question structure prior to survey distribution. The survey was distributed via email on 14 November 2018 and remained accessible through 13 December 2018, with a reminder email sent to potential participants on 30 November 2018. Respondents were eligible to enter a raffle for one of ten $25 gift cards. This study was reviewed and deemed exempt by the Institutional Review Board at the University of North Carolina at Chapel Hill.

Responses to questions utilizing Likert scales were dichotomized for the analysis. Positive responses (e.g., agree and strongly agree; somewhat comfortable and very comfortable) and, separately, negative or neutral responses, were combined to indicate either a positive or negative/neutral response to the question. Descriptive statistics characterized the demographic characteristics of the survey respondents and their responses. Chi-square tests were used to detect differences in demographics, previous training/education, and agreement with perceived benefits of pharmacist-prescribed hormonal contraception between community and noncommunity pharmacists. Data were analyzed in SAS v9.4 (SAS Institute, Cary, NC, USA).

## 3. Results

### 3.1. Demographics and Baseline Characteristics

Of the 12,001 actively licensed pharmacists residing in North Carolina surveyed, 713 (5.9%) responded. Of these, 384 (54.2%) identified as working in a community-based setting, while the remainder endorsed working in a noncommunity setting (i.e., inpatient, ambulatory care, industry, etc.) (Table 1). Overall, respondents were generally female, younger than 40 years old, licensed for 10 or less years, and had a Doctor of Pharmacy (PharmD) degree. In total, 670 respondents provided information on their location. Of those, 208 (29.2%) respondents reported working in an urban location, 271 (38%) in a suburban location, and 191 (26.8%) in a rural location. Notably, 43 (6%) participants selected “prefer not to answer” to this question. See Table 1 for additional details regarding demographic characteristics of participants.

Community pharmacy characteristic data were compiled in this survey in order to describe and evaluate the current clinical practices and environment of privacy that community pharmacists note in their setting. Among community pharmacists, 88 out of 360 (22.9%) reported that they considered the counseling area within their pharmacy “private”. The most common clinical services available at respondent’s pharmacies were administering immunizations (77.6%), sale of emergency contraception (76.3%), and medication therapy management (53.9%).

### 3.2. Perceived Benefits and Barriers of Pharmacist-Prescribed Hormonal Contraception

When asked to rate the extent of agreement to five statements regarding the benefits of pharmacist-prescribed contraception, several statistically significant differences were noted between community and noncommunity pharmacists (Table 2). Noncommunity pharmacists were more likely to agree that prescribing hormonal contraception allows pharmacists to practice at a higher level (81.4% vs 66.9%; *p* = 0.02). Noncommunity pharmacists were also more likely to agree that rural areas would benefit from pharmacist-prescribed hormonal contraception (81.2% vs 66.7%; *p* = 0.02). Finally, noncommunity pharmacists were more likely to agree that increased access to hormonal contraception is an important public health issue (78.4% vs 64.8%; *p* = 0.04).

The majority of community pharmacists felt that pharmacist-prescribed contraception would increase use and adherence (69.5%) and that patients would benefit from improved access (68.8%). More than half of respondents (53.4%) believed that prescribing contraception would increase business and revenue for the pharmacy; only 13.3% felt that prescribing would help recruit pharmacists to work in their store. Roughly half of noncommunity pharmacists felt that there are significant barriers to pharmacist-prescribed hormonal contraception within community pharmacies (50.5%), and fewer than half (44.1%) felt there would be high acceptance of prescribing hormonal contraception among community pharmacists (Table 3).

The five most common barriers to prescribing contraception noted by community-based pharmacists were added responsibility and liability (69.8%), time constraints (67.2%), need for pharmacist training (65.4%), resistance from physicians (56.3%), and reimbursement barriers (54.4%). Less than one-fifth of respondents felt that resistant from management and resistance from patients would be significant barriers.

### 3.3. Pharmacist Interest and Comfort Level in Prescribing

**Figure 1:**
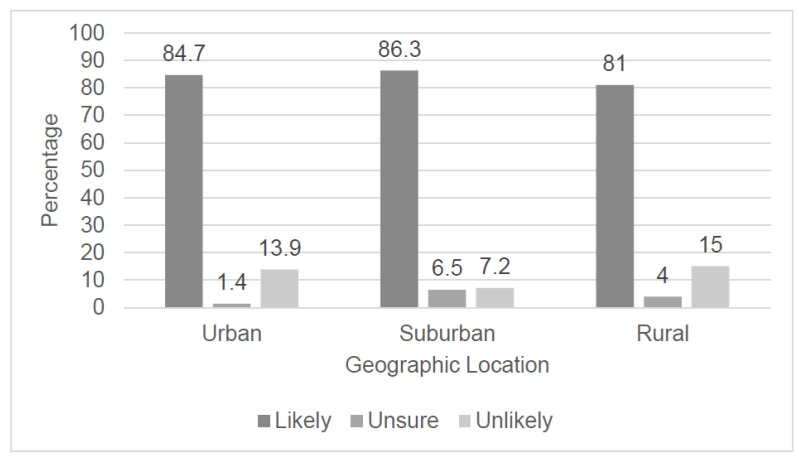
Likelihood to prescribe hormonal contraception if all barriers are removed and adequate training is provided, stratified by geographic location of respondents’ pharmacy practice, *n* = 305 (*p* = 0.55).

Overall, 82.6% of community pharmacists surveyed in North Carolina reported that they were likely to prescribe, assuming that all barriers were removed and sufficient training was provided. When stratified by geographic location, no statistically significant difference existed in likelihood to prescribe between respondents self-reporting a primary practice site in either rural, suburban, or urban areas (81.0% vs 86.3% vs 84.7%, respectively; *p* = 0.55) (Figure 1).

### 3.4. Pharmacist Opinion of Prescriptive Classification of Hormonal Contraceptives

A slight majority of all respondents (51.1%) believed that hormonal contraception should be classified as pharmacist-prescribed, followed by a quarter (25.4%) believing it should be prescription only. Other classification responses were as follows: 15.6% believed it should be classified as behind-the-counter, without a prescription; 4.2% over-the-counter, with no age restrictions; and 3.7% over-the-counter, with age restrictions.

## 4. Discussion

This study sought to determine the likelihood of community pharmacists to prescribe hormonal contraception, identify potential barriers, and to understand the perceptions of all pharmacists regarding this practice. Overall, community pharmacists in North Carolina indicated a high likelihood that they would support hormonal contraception prescribing if all barriers were removed and training were provided. These data are important given the role that pharmacists may have in increasing access to contraception in a state where 90% of the counties are designated as Primary Care-Health Professional Shortage Areas [10]. The prevalence and accessibility of pharmacists in North Carolina continues to increase. Recent data show that, compared to other types of health professionals such as physicians, pharmacists are fairly evenly distributed between metropolitan and nonmetropolitan counties in NC, with 71 counties endorsing an increase in number of pharmacists from 2008 to 2012 [12]. It is important to note that our results showed no significant difference between support of prescribing amongst rural and nonrural pharmacists. This shows that community pharmacies are well-positioned to address disparities in access to healthcare services, including contraceptive access. Additionally, this survey was the first to be completed in the southeastern region of the country. The knowledge gained from this study will allow researchers to determine overall interest in pursuing legislation on the state level, but may apply to other areas within our region, where significant support and advocacy from all pharmacists is necessary to pass legislature.

In this study, the most frequently noted barriers included added responsibility and liability, time constraints, need for pharmacist training, resistance from physicians, and reimbursement barriers. These were consistent with previous investigations [13,14]. A previous study completed in California one year after pharmacist-prescribed hormonal contraception legislation was implemented showed that existing barriers may directly impact uptake of services. Specifically, this study found that a low uptake of services (11.1%) was primarily attributed to barriers such as payment for pharmacy services [15]. Thus, it is known that understanding existing barriers is imperative to successful enactment. Understanding potential barriers will inform policymakers; should North Carolina pursue legislation, it will be possible to proactively identify solutions. Potential solutions to the currently identified barriers include a training program to increase comfort level with prescribing, ongoing discussions with major payors in the state for reimbursement purposes, and the development of protocols and algorithms that allow pharmacists to quickly identify patients who are candidates for prescribing and those that warrant a referral as well as address potential safety concerns. As previous studies noted similar barriers, in 2019, Rafie et al. discussed potential solutions to many of these barriers, including ongoing education to alleviate safety concerns, importance of technology changes, barriers related to pharmacy space and logistics, and continued training to provide support to pharmacists [16]. Additionally, it is known that addressing reimbursement and payment-for-service is important to enacting this practice, as in states that do not have a process in place for payment, uptake of the practice may be stalled [15]. As our study did not evaluate these potential solutions to barriers, further investigation is needed in subsequent research.

Support of hormonal contraception prescribing among community pharmacists in North Carolina was greater than what was noted in similar, previous surveys conducted in other states. These studies showed that relative intent to prescribe was 57–72.7%, assuming that adequate training and reimbursement were provided [13,14]. The greater support observed in this study may be attributed to differing language of the question or to more significant enthusiasm from respondents of this survey as compared to the general pharmacist population. It is also known that responses do not necessarily translate to clinical practice. Multiple states gauged a much larger initial enthusiasm for this practice than actual implementation, which was largely attributed to financial and logistical barriers [15,17]. However, even with lower-than-expected rates of adoption of this practice, economic and public health impact can still be achieved. Oregon reported that, in the first two years of their program, 10% of all new oral and transdermal contraceptive Medicaid prescriptions were written by pharmacists, 74% of which were written to patients who had not used birth control in the previous month [17]. Additionally, it was estimated that more than 50 unintended pregnancies were avoided, translating to an estimated $1.6 million in public cost savings [18].

This study also examined if differences existed between community and noncommunity pharmacists regarding the perception of potential benefits of pharmacist-prescribed hormonal contraception. The findings indicate that noncommunity pharmacists were significantly more likely to agree that rural areas would benefit from pharmacist-prescribed hormonal contraception, prescribing hormonal contraception allows pharmacists to practice at a higher level, and increased access to hormonal contraception is an important public health issue. One explanation for this finding may be concern by community pharmacists for the increased workload. As noted in several survey comments, community pharmacists felt that prescribing hormonal contraception will simply become a monetized service, added to a long list of tasks to be completed daily, and not a pathway to practicing at a higher level or providing needed services to the community. This finding illustrates the need to address these concerns with adequate resources and training to expedite the prescribing process. However, the support of this practice amongst all pharmacists in the state is important as advocacy for its enactment often occurs on the state level, through state organizations, and unification of all pharmacist voices advocating for the same practice advancement is crucial.

Finally, this study notably found that fewer than 10% of respondents felt that hormonal contraception should be classified as “over-the-counter (OTC)”. This remains consistent with a previous study that found that 78% of pharmacists surveyed nationwide opposed the OTC status of combined hormonal contraception [19]. The American College of Obstetrics and Gynecology (ACOG), the American Medical Association (AMA), and the American Academy of Family Physicians (AAFP) all recommend that oral contraceptives be available OTC in order to increase contraceptive access, indicating a divergence with professional medical organizations [6]. In this survey, the pharmacists surveyed were able to select only one classification for hormonal contraception. The majority selected “pharmacist-prescribed”; however, it is possible that respondents may have selected additional classifications, including “OTC”, provided the opportunity. However, given that no current OTC contraceptive exists and the barriers to the FDA approval of one, pharmacist-prescribed hormonal contraception is one of the most prominent methods available to increase access to not only oral hormonal contraception, but nonoral forms as well [20].

### Limitations

Several limitations to this study exist. First, the response rate of this survey was low. Other initial interest surveys conducted in California and Oregon to all pharmacists had response rates of 14.5% and 17%, respectively, indicating that low response rates are typical for this type of survey [10,11,12]. Our particularly low response rate may be attributed to the timing of survey distribution around holidays in November and December. It also may be attributed to the larger and more diverse target population of this study, given that we surveyed all pharmacists residing in North Carolina, not simply those currently practicing or those only in community pharmacy settings. It is also possible that noncommunity pharmacists did not feel qualified or have interest in responding to this survey. Thus, the results of this survey may not be generalizable to all North Carolina pharmacists or those practicing in other states.

Similarly, responses in this research do not necessarily correlate to interest in the state as this survey represented a sample of convenience. Those who had stronger opinions and interest in the survey as well as those who are working to advance community practice may have been more likely to respond. This may result in a nonresponse bias that created a higher rate of pharmacist interest than exists within the state as a whole. A modest incentive was offered for completing the survey, which may have attracted respondents less interested or indifferent to the subject matter to address the potential of nonresponse bias. As a result, the responses herein may not be representative of all North Carolina pharmacists; however, research from Oregon shows that, even with a low response rate, interest may still correlate to likelihood of enactment and practice [13].

Additionally, it is important to note that a validated theoretical framework was not utilized in the creation of this study. As a result, it is possible that all factors were not considered in studying the association of pharmacist-prescribed hormonal contraception. Future research should explore this using practice behavior or implementation frameworks. Finally, based on zip code information provided by respondents, only 81 out of 100 counties had representation within the survey. However, 150 respondents chose not to enter their zip code. This may have been out of concern for compromising anonymity as fewer pharmacy practice sites exist within certain zip codes, particularly in rural counties of North Carolina where an underrepresentation of zip codes was noted. Additionally, the number of respondents skewed toward counties located in more urban areas with larger populations. This may limit generalizability to support and interest in rural counties of North Carolina.

## 5. Conclusions

Assuming that all barriers are removed, and sufficient training is provided, most community pharmacist respondents in North Carolina are likely to prescribe hormonal contraception. Limitations to this study include a low response rate and a convenience sample of pharmacist respondents. This research supports pharmacy practice advancement to authorize pharmacist-prescribed hormonal contraception among community pharmacists. Future directions include identifying proficient policies to overcome barriers to implementation.

## Figures and Tables

**Table 1:** Baseline demographics, stratified by primary practice site.

	Total, N (%)	Community-Based Pharmacist, N (%)	Non Community Pharmacist, N (%)	*p* Value
All Pharmacist Characteristics (*n* = 713)				
**Age**				
<40 years old	372 (52.1)	207 (55.6)	165 (44.4)	0.07
40–59 years old	220 (30.9)	101 (45.9)	119 (54.1)	
≥60 years old	102 (14.3)	52 (51)	50 (49)	
Missing	19 (2.7)			
**Gender**				
Female	462 (64.8)	224 (48.5)	238 (51.5)	0.01
Missing	13 (1.8)			
**Years as Licensed Pharmacist**				
≤10 years	340 (47.7)	196 (57.6)	144 (42.4)	0.01
11–20 years	122 (17.1)	59 (48.4)	63 (51.6)	
≥20 years	242 (33.9)	110 (45.5)	132 (54.5)	
Missing	9 (1.3)			
**Pharmacy Education/Training**				
Doctor of Pharmacy (PharmD) degree	515 (72.2)	260 (50.5)	255 (49.5)	0.23
Post-Graduate Year 1 (PGY1) Residency	122 (17.1)	14 (11.5)	108 (88.5)	<0.0001
Missing	5 (7)			
**Previous Education/Training**				
Reviewed prescribing protocols during pharmacy school	298 (41.8)	156 (52.3)	142 (41.9)	0.82
Continuing Education course	229 (32.7)	126 (55) (55.1)	103 (45)	0.25
None	308 (43.2)	153 (49.7)	155 (50.3)	0.31
Other	15 (2.1)	8 (53.3)	7 (46.7)	0.91
Missing	13 (1.8)			
**Geographic Location of Practice Site**				
Urban	208 (29.2)	88 (42.3)	120 (57.7)	0.0002
Suburban	271 (38)	152 (56.1)	119 (43.9)	
Rural	191 (26.8)	119 (62.3)	72 (37.7)	
Missing	43 (6)			
**Primary Practice Site ^a^**				
Community Practice—Chain	251 (35.2)	251 (35.2)	--	N/A
Community Practice—Independent	118 (16.5)	118 (16.5)	--	
Community Pharmacy Owner	15 (2.1)	15 (2.1)	--	
Clinical Pharmacist—Hospital	92 (12.9)	--	92 (12.9)	
Clinical Pharmacist—Ambulatory Care	64 (9)	--	64 (9)	
Staff Hospital Pharmacist	57 (8)	--	57 (8)	
Industry	28 (3.9)	--	28 (3.9)	
Long-Term Care Pharmacy	26 (3.6)	--	26 (3.6)	
Hospital Pharmacy Administration	25 (3.5)	--	25 (3.5)	
Academia	22 (3.1)	--	22 (3.1)	
Missing	4 (0.6)			
**Community Pharmacy Characteristics (*n* = 384)**				
**Clinical Services Offered by Pharmacy**				
Immunizations	298 (77.6)	298 (77.6)	--	N/A
Medication Therapy Management	207 (53.9)	207 (53.9)	--	N/A
Missing	49 (12.8)			
**Emergency Contraception Available for Sale**				
Yes	293 (76.3)	293 (76.3)	--	N/A
Missing	23 (6)			
**Privacy of Counseling Area**				
Private	88 (22.9)	88 (22.9)	--	N/A
Semi-private	154 (40.1)	154 (40.1)	--	
Not private	115 (29.9)	115 (29.9)	--	
Missing	24 (6.3)			

^a^ respondents could select multiple practice sites.

**Table 2:** Agreement with perceived benefits of pharmacist-prescribed hormonal contraception, stratified by primary practice site.

Statement	Community-Based Pharmacists (*n* = 384), N (%)	Noncommunity Pharmacists (*n* = 329), N (%)	*p* Value
Pharmacists are well-trained/educated to prescribe hormonal contraception.	149 (38.8)	160 (48.6)	0.15
Prescribing hormonal contraception allows pharmacists to practice at a higher level.	257 (66.9)	268 (81.4)	0.02
Increased access to hormonal contraception is an important public health issue.	249 (64.8)	258 (78.4)	0.04
Prescribing hormonal contraception will strengthen relationships with local physicians and clinics.	138 (35.9)	116 (35.3)	0.18
Rural areas would benefit from pharmacist-prescribed hormonal contraception.	256 (66.7)	267 (81.2)	0.02
Prescribing hormonal contraception will increase business/revenue in my pharmacy.	205 (53.4)	--	N/A
Prescribing hormonal contraception will help recruit pharmacists to work in our store.	51 (13.3)	--	N/A
Patients will benefit from improved access to hormonal contraception.	264 (68.8)	--	N/A
As a pharmacist, I enjoy individual patient contact.	300 (78.1)	--	N/A
Pharmacy access to hormonal contraception may foster increased use and adherence.	267 (69.5)	--	N/A
There are significant barriers to pharmacist-prescribed hormonal contraception within community pharmacies.	--	166 (50.5)	N/A
Additional training or education should be required for pharmacists to prescribe hormonal contraceptives.	--	273 (83)	N/A
There would be high acceptance of prescribing hormonal contraception amongst community pharmacists.	--	145 (44.1)	N/A

**Table 3:** Extent of agreement among community pharmacists with perceived barriers to pharmacist-prescribed hormonal contraception.

Barrier (*n* = 384)	Community-Based Pharmacists, N (%)
Added responsibility and liability	268 (69.8)
Time constraints	258 (67.2)
Need for pharmacist training	251 (65.4)
Resistance from physicians	216 (56.3)
Reimbursement barriers	209 (54.4)
Inadequate privacy for counseling	190 (49.5)
Pharmacist disinterest in prescribing hormonal contraception	150 (39.1)
Resistance from management	76 (19.8)
Resistance from patients	61 (15.9)

## Supplementary Materials

The following are available online at https://www.mdpi.com/2226-4787/8/4/191/s1, Survey: Perceptions Regarding Pharmacist Prescribed Hormonal Contraception.
