# Supplementary material for: Role of Pharmacists in Hormonal Contraceptive Access: A Survey of North Carolina Pharmacists
Source: Pharmacy (Basel). 2020 Oct 16;8(4):191. doi: 10.3390/pharmacy8040191 (PMC7712350; doi:10.3390/pharmacy8040191)
Supplement: Supplementary file 1 [file pharmacy-08-00191-s001.pdf]

Perceptions Regarding Pharmacist Prescribed Hormonal Contraception

Start of Block: Informed Consent

Q32

Welcome!

Thank you for participating in the Perceptions Regarding Pharmacist Prescribed Hormonal Contraception survey. Your participation is voluntary and your answers are completely confidential. You may skip any question you choose not to answer.

At the end of survey, if you wish to be entered into a drawing to win one of ten $25 Amazon gift cards, please provide your name and email address. These will be kept separate and unlinked from the data submitted in the survey, keeping all responses anonymous.

Please indicate your consent below and continue on to begin the survey.

○ I consent, begin the study (1)

○ I do not consent, I do not wish to participate (2)

End of Block: Informed Consent

Start of Block: Demographics

Q17 Unique Identifier: First 3 letters of middle name and last 4 digits of cell phone number (Ex: JEA7212)

____

Q1 What is your age?

- ○ Younger than 30 years old (1)
- ○ 30-39 years old (2)
- ○ 40-49 years old (3)
- ○ 50-59 years old (4)
- ○ 60 years or older (5)
- ○ Prefer not to answer (6)

Q2 What is your gender?

- ○ Male (1)
- ○ Female (2)
- ○ Prefer not to answer (3)

Q3 How many years have you been a licensed pharmacist?

- ○ Less than 1 year (7)
- ○ 1-5 years (8)
- ○ 6-10 years (9)
- ○ 11-15 years (10)
- ○ 16-20 years (11)
- ○ Greater than 20 years (12)

Q6 What pharmacy education, training, and/or certifications have you completed or are in progress to complete? (Select all that apply.)

- ▢ PharmD (1)
- ▢ BS Pharmacy (2)
- ▢ PGY1 Community-based Pharmacy Residency (3)
- ▢ PGY1 Managed Care Pharmacy Residency (4)
- ▢ PGY1 Pharmacy Residency (5)
- ▢ PGY2 (6)
- ▢ Fellowship (7)
- ▢ MS (8)
- ▢ PhD (9)
- ▢ Board Certified Ambulatory Care Pharmacy (BCACP) (10)
- ▢ Board Certified Pharmacotherapy Specialists (BCPS) (11)
- ▢ Other (12) ____
- ▢ None (13)

Q7 From what state did you graduate pharmacy school?
Select state (1)

▼ AL (1) ... WY (51)

Q27 What education and/or training have you received in prescribing hormonal contraception

- ☐ Reviewed prescribing protocols during pharmacy school (1)
- ☐ APhA's Advanced Training Program in "Increasing Access to Hormonal Contraceptive Products" (2)
- ☐ Through state association program (3)
- ☐ Continuing Education course (4)
- ☐ None (5)
- ☐ Other (6) ____

Q5 What is your primary pharmacy practice? (Select all that apply)

- ☐ Community Practice- Chain (1)
- ☐ Community Practice - Independent (14)
- ☐ Community Pharmacy Owner (3)
- ☐ Hospital Pharmacy Administration (2)
- ☐ Staff Hospital Pharmacist (5)
- ☐ Clinical Pharmacist- Hospital (6)
- ☐ Clinical Pharmacist- Ambulatory Care (7)
- ☐ Academia (8)
- ☐ Nuclear Pharmacy (9)
- ☐ Managed Care Pharmacy (10)
- ☐ Long-term Care Pharmacy (11)
- ☐ Mail-order pharmacy (12)
- ☐ Industry (15)
- ☐ Other (13) ____

Q16 In what zip code is your primary pharmacy practice site located?

- ○ Please enter your zip code (1) ____
- ○ Prefer not to answer (2)

Q28 How would you classify the geographic location of your primary pharmacy site?

- Urban (1)
- Suburban (2)
- Rural (3)
- Prefer not to answer (4)

Display This Question:

If What is your primary pharmacy practice? (Select all that apply) = Community Practice- Chain

Or What is your primary pharmacy practice? (Select all that apply) = Community Pharmacy Owner

Or What is your primary pharmacy practice? (Select all that apply) = Community Practice - Independent

Q9 What clinical services are offered by your pharmacy?

- ▢ Asthma Management (1)
- ▢ Osteoporosis management (2)
- ▢ Diabetes management (3)
- ▢ Emergency contraception (4)
- ▢ Hypertension management (5)
- ▢ Immunizations (6)
- ▢ Lipid management (7)
- ▢ Medication therapy management (8)
- ▢ Smoking cessation (9)
- ▢ Travel medicine (10)
- ▢ Weight management (11)
- ▢ Specialty Pharmacy (13)
- ▢ Other (12) ____

Display This Question:

If What is your primary pharmacy practice? (Select all that apply) = Community Practice- Chain

Or What is your primary pharmacy practice? (Select all that apply) = Community Practice - Independent

Or What is your primary pharmacy practice? (Select all that apply) = Community Pharmacy Owner

Q29 What would you consider the level of privacy of your pharmacy's counseling area?

○ Private (1)

○ Semi-private (2)

○ Not private (3)

Display This Question:

If What is your primary pharmacy practice? (Select all that apply) = Community Practice- Chain

Or What is your primary pharmacy practice? (Select all that apply) = Community Pharmacy Owner

Or What is your primary pharmacy practice? (Select all that apply) = Community Practice - Independent

Q11 Is emergency contraception available for sale in your pharmacy?

○ Yes (1)

○ No (2)

○ Unsure (3)

End of Block: Demographics

Start of Block: Determining Likelihood to Participate

Display This Question:

If What is your primary pharmacy practice? (Select all that apply) = Community Practice- Chain

Or What is your primary pharmacy practice? (Select all that apply) = Community Pharmacy Owner

Or What is your primary pharmacy practice? (Select all that apply) = Community Practice - Independent

Q13 Please indicate below the extent of your agreement with the following statements

	Strongly disagree (1)	Disagree (2)	Undecided (3)	Agree (4)	Strongly Agree (5)

Patients will benefit from improved access to hormonal contraception (1)	○	○	○	○	○
Pharmacists are well-trained/educated to prescribe hormonal contraception (2)	○	○	○	○	○
Pharmacy access to hormonal contraception may foster increased use and adherence (3)	○	○	○	○	○
Prescribing hormonal contraception allows pharmacists to practice at a higher level (4)	○	○	○	○	○
Increased access to hormonal contraception is an important public health issue (5)	○	○	○	○	○
As a pharmacist, I enjoy individual patient contact (6)	○	○	○	○	○
Prescribing hormonal contraception will strengthen relationships with local physicians and clinics (7)	○	○	○	○	○

Prescribing hormonal contraception will increase business/revenue in my pharmacy (8)	o	o	o	o	o
Prescribing hormonal contraception will help recruit pharmacists to work in our store (9)	o	o	o	o	o
Rural areas would benefit from pharmacist-prescribed hormonal contraception (10)	o	o	o	o	o

Display This Question:

If What is your primary pharmacy practice? (Select all that apply) = Community Practice- Chain

Or What is your primary pharmacy practice? (Select all that apply) = Community Practice - Independent

Or What is your primary pharmacy practice? (Select all that apply) = Community Pharmacy Owner

Q18 Please list other reasons that may increase your likelihood to prescribe hormonal contraception, if allowed within the scope of practice in North Carolina

____

Display This Question:

If What is your primary pharmacy practice? (Select all that apply) = Community Practice- Chain

Or What is your primary pharmacy practice? (Select all that apply) = Community Pharmacy Owner

Or What is your primary pharmacy practice? (Select all that apply) = Community Practice - Independent

Q19 Would any of the following hinder you from prescribing hormonal contraception if it was allowed within the scope of practice in North Carolina? (Select all that apply.)

- ☐ Personal Beliefs (1)
- ☐ Religious Beliefs (2)
- ☐ Conflicts of interest (i.e. personal relationships with patients outside of pharmacy) (3)
- ☐ None (4)
- ☐ Other (5) ____

Display This Question:

If What is your primary pharmacy practice? (Select all that apply) = Community Practice- Chain

Or What is your primary pharmacy practice? (Select all that apply) = Community Pharmacy Owner

Or What is your primary pharmacy practice? (Select all that apply) = Community Practice - Independent

Q14 Please indicate below the extent to which you believe the following barriers will impact your ability to prescribe hormonal contraception in the pharmacy

	Strongly Disagree (1)	Disagree (2)	Undecided (3)	Agree (4)	Strongly Agree (5)

Time constraints (1)	○	○	○	○	○
Provision of hormonal contraception will result in added responsibility and liability (2)	○	○	○	○	○
A pelvic exam and pap smear are necessary for safe prescribing (3)	○	○	○	○	○
Resistance from management (4)	○	○	○	○	○
Resistance from patients (5)	○	○	○	○	○
Resistance from physicians (6)	○	○	○	○	○
Pharmacist disinterest in prescribing hormonal contraception (7)	○	○	○	○	○
Reimbursement barriers (8)	○	○	○	○	○
Need for pharmacist training (9)	○	○	○	○	○
Inadequate privacy for counseling (10)	○	○	○	○	○

Display This Question:

If What is your primary pharmacy practice? (Select all that apply) = Community Practice- Chain

Or What is your primary pharmacy practice? (Select all that apply) = Community Pharmacy Owner

Or What is your primary pharmacy practice? (Select all that apply) = Community Practice - Independent

Q16 What other barriers, if any, do you identify in prescribing hormonal contraception?

____

Display This Question:

If What is your primary pharmacy practice? (Select all that apply) != Community Practice- Chain

And What is your primary pharmacy practice? (Select all that apply) != Community Pharmacy Owner

And What is your primary pharmacy practice? (Select all that apply) != Community Practice - Independent

Q17 Please indicate below the extent of your agreement with the following statements

	Strongly Disagree (1)	Disagree (2)	Undecided (3)	Agree (4)	Strongly Agree (5)

There are significant barriers to pharmacist-prescribing hormonal contraception within community pharmacies (1)	○	○	○	○	○
Pharmacists are well-trained/educated to prescribe hormonal contraception (2)	○	○	○	○	○
Additional training or education should be required for pharmacists to prescribe hormonal contraceptives (3)	○	○	○	○	○
There would be high acceptance of prescribing hormonal contraception amongst community pharmacists (4)	○	○	○	○	○
Increased access to hormonal contraception is an important public health issue (5)	○	○	○	○	○

Rural patients would benefit from pharmacist-prescribed hormonal contraception (6)	○	○	○	○	○
Prescribing hormonal contraception will strengthen relationships between local pharmacies and physicians (7)	○	○	○	○	○
Prescribing hormonal contraception allows pharmacists to practice at a higher level (8)	○	○	○	○	○

End of Block: Determining Likelihood to Participate

Start of Block: Additional Training Requested

Display This Question:

If What is your primary pharmacy practice? (Select all that apply) = Community Practice- Chain

Or What is your primary pharmacy practice? (Select all that apply) = Community Pharmacy Owner

Or What is your primary pharmacy practice? (Select all that apply) = Community Practice - Independent

Q20 Please rate the extent of your comfort in providing counseling for patients on proper hormonal contraception use with the following products.

	Very Uncomfortable (1)	Somewhat Uncomfortable (2)	Neither Comfortable nor Uncomfortable (3)	Somewhat Comfortable (4)	Very Comfortable (5)
Oral (1)	○	○	○	○	○
Transdermal (i.e. Ortho Evra) (2)	○	○	○	○	○
Injection (i.e. Depo-Provera) (3)	○	○	○	○	○
Intra-vaginal (NuvaRing) (4)	○	○	○	○	○

Display This Question:

If What is your primary pharmacy practice? (Select all that apply) = Community Practice- Chain

Or What is your primary pharmacy practice? (Select all that apply) = Community Pharmacy Owner

Or What is your primary pharmacy practice? (Select all that apply) = Community Practice - Independent

Q21 States with pharmacist-prescribed hormonal contraception have required training/continuing education credits prior to participating in the program. If training were provided, how comfortable would you feel prescribing hormonal contraception?

- ○ Extremely comfortable (1)
- ○ Somewhat comfortable (2)
- ○ Neither comfortable nor uncomfortable (3)
- ○ Somewhat uncomfortable (4)
- ○ Extremely uncomfortable (5)

End of Block: Additional Training Requested

Start of Block: These questions will address hormonal contraception knowledge

Q22 Rate each of the following statements as true or false.

	True (1)	False (2)
Combined hormonal contraception is contraindicated less than 21 days postpartum (1)	○	○
If a dose of progestin-only contraceptive is taken 6 hours late, it is considered a missed dose (2)	○	○
In a patient being initiated on lamotrigine, combined hormonal contraception may cause increased levels of lamotrigine (3)	○	○
Increasing the estrogen content of combined hormonal contraception can help with breakthrough bleeding in days 1-9 (4)	○	○
NuvaRing can be left in for 28 days and the women will remain protected against unintended pregnancy (5)	○	○
Progestin-only contraceptives can be used in patients with active breast cancer (6)	○	○
Oral combined hormonal contraception is contraindicated in females over 35 that smoke >15 cigarettes/day (7)	○	○
Oral combined hormonal contraception is contraindicated in females with history of migraine with aura (8)	○	○

End of Block: These questions will address hormonal contraception knowledge

Start of Block: The following questions will address prescription vs. over-the-counter status

Q23 The American Medical Association (AMA), American Academy of Family Physicians (AAFP), and American College of Obstetricians and Gynecologists (ACOG) support making oral hormonal contraception available over-the-counter. Of the following medication statuses, in which do you believe oral hormonal contraception should be categorized?

- Prescription only (1)
- Pharmacist-prescribed (2)
- Behind-the-counter, without a prescription (3)
- Over-the-counter with age restrictions (4)
- Over-the-counter with no age restrictions (5)

Display This Question:

If What is your primary pharmacy practice? (Select all that apply) = Community Practice- Chain

Or What is your primary pharmacy practice? (Select all that apply) = Community Pharmacy Owner

Or What is your primary pharmacy practice? (Select all that apply) = Community Practice - Independent

Q24 If allowed as a scope of practice for pharmacists in North Carolina, how comfortable would you be prescribing and dispensing hormonal contraception if it was available through the following procedures

	Very uncomfortable (1)	Somewhat uncomfortable (2)	Neither comfortable nor uncomfortable (3)	Somewhat comfortable (4)	Very comfortable (5)
Under state-wide protocol or standing order (1)	○	○	○	○	○
Under a collaborative practice agreement (2)	○	○	○	○	○
Over-the-counter with regulations (3)	○	○	○	○	○
Over-the-counter with no regulations (4)	○	○	○	○	○

Display This Question:

If What is your primary pharmacy practice? (Select all that apply) = Community Practice- Chain

Or What is your primary pharmacy practice? (Select all that apply) = Community Pharmacy Owner

Or What is your primary pharmacy practice? (Select all that apply) = Community Practice - Independent

Q25 Assuming all barriers were removed and sufficient training were provided, how likely would you be to prescribe hormonal contraception in North Carolina?

- ◯ Extremely likely (1)
- ◯ Moderately likely (2)
- ◯ Slightly likely (3)
- ◯ Neither likely nor unlikely (4)
- ◯ Slightly unlikely (5)
- ◯ Moderately unlikely (6)
- ◯ Extremely unlikely (7)

Q26 Please leave any comments/suggestions below you may have regarding pharmacist-prescribing hormonal contraception in North Carolina

____

End of Block: The following questions will address prescription vs. over-the-counter status

---

Start of Block: Block 6
